# Supplementary material for: Lysosomal damage drives mitochondrial proteome remodelling and reprograms macrophage immunometabolism
Source: Nat Commun. 2022 Nov 28;13:7338. doi: 10.1038/s41467-022-34632-8 (PMC9705561; doi:10.1038/s41467-022-34632-8)
Supplement: Supplementary file 1 — Supplementary Information [file 41467_2022_34632_MOESM1_ESM.pdf]

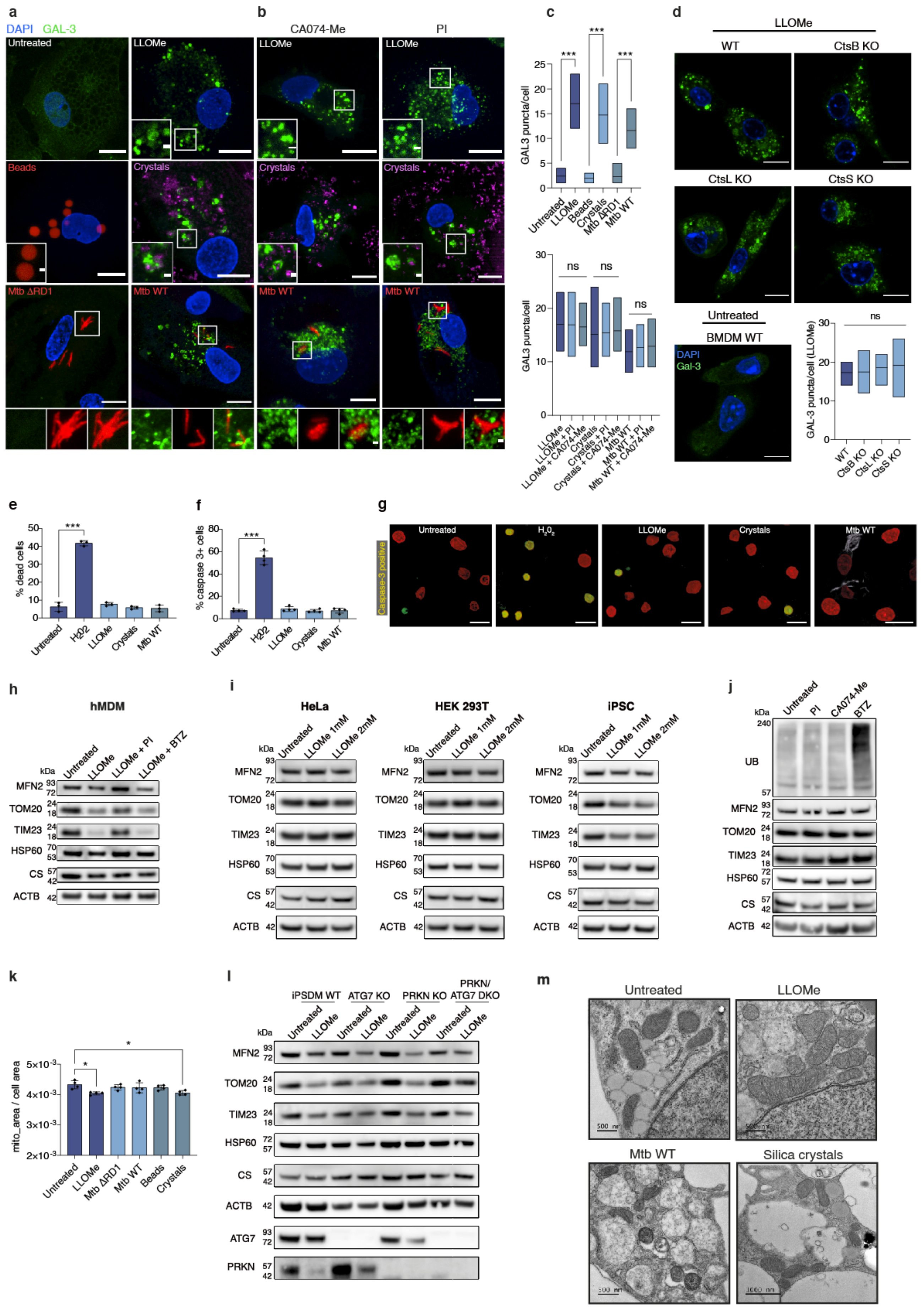
a
DAPI GAL-3
Untreated
LLOMe
Beads
Crystals
Mtb ΔRD1
Mtb WT
b
CA074-Me
PI
LLOMe
Crystals
Mtb WT
c
GAL3 puncta/cell
Untreated
LLOMe
Beads
Crystals
Mtb ΔRD1
Mtb WT
***
ns
LLOMe + PI
LLOMe + CA074-Me
Crystals + PI
Crystals + CA074-Me
Mtb WT + PI
Mtb WT + CA074-Me
d
LLOMe
WT
CtsB KO
CtsL KO
CtsS KO
Untreated
BMDM WT
DAPI
Gal-3
GAL-3 puncta/cell (LLOMe)
e
% dead cells
H2O2
f
% caspase 3+ cells
g
Caspase-3 positive
h
hMDM
LLOMe + PI
LLOMe + BTZ
kDa
MFN2
TOM20
TIM23
HSP60
CS
ACTB
UB
i
HeLa
HEK 293T
iPSC
LLOMe 1mM
LLOMe 2mM
j
BTZ
k
mito_area / cell area
l
iPSDM WT
ATG7 KO
PRKN KO
PRKN/ ATG7 DKO
ATG7
PRKN
m
Silica crystals

Supplementary Figure 1- Supplementary data related to Figure 1.

(a) iPSDM stimulated with 0.5 mM LLOMe for 1 h, 100 ug/mL silica crystals or beads for 3 h or infected with Mtb WT or Mtb ΔRD1 for 48 h. n = 3 independent experiments **(b)** Same conditions as in (a) but co-treated with the indicated protease inhibitors. **(c)** Quantitative analysis of Galectin-3 (Gal-3) puncta by confocal imaging. At least 40 cells were counted per condition. **(d)** BMM WT, CtsB KO, CtsL KO and CtsS KO were stimulated with 0.5 mM LLOMe for 1 h and Gal-3 puncta evaluated by confocal imaging. At least 30 cells were counted per condition. **(e, f)** iPSDM were stimulated with 0.5mM LLOMe for 1 h, 100 ug/mL silica crystals for 3 h or infected with Mtb WT for 48 h and cellular viability evaluated using a Cell Viability Imaging Kit (e) or a Caspase-3 assay kit (f). 1 mM H_2O_2 for 1h was used as a positive control. **(g)** Representative images from one out of three independent experiments showing iPSDM stained with Caspase-3 assay kit (Caspase-3+ nuclei shown in yellow). (**h**) Immunoblot for MFN2, TOM20, TIM23, HSP60 and Citrate synthase (CS) in primary human blood monocyte-derived macrophages left untreated or treated with 0.5 mM LLOMe for 1h in presence or absence of PI or BTZ. ACTB levels were used as loading controls (repeated three times with similar results). (**i**) Immunoblot for mitochondrial proteins in HeLa, HEK 293T cells and Eika2 iPSC treated or not with LLOMe for 6 h at the indicated concentrations (repeated three times with similar results). **(j)** Immunoblot for total ubiquitin (UB) and mitochondrial proteins in iPSDM incubated in the presence or absence of 1:400 protease inhibitors, 50 uM CA074-Me, or 5 nM bortezomib for 1 h (repeated three times with similar results). **(k)** Quantitative analysis of mitochondrial area normalized to cellular area in iPSDM after the indicated conditions. At least 700 cells were counted per condition. **(l)** Western blot analysis of mitochondrial proteins in iPSDM WT, ATG7 KO, PRKN KO and PRKN/ATG7 DKO stimulated with 0.5 mM LLOMe for 1 h. Actin (ACTB) levels were used as loading controls. **(m)** Representative electron micrographs of Mtb WT-infected, silica crystals and LLOMe-treated macrophages. No mitochondrial derived vesicles (MDV) were detected. Mitochondria counted per condition from at least 19 cells; untreated: 149, LLOMe-treated: 121, Mtb WT infected: 177 and Silica crystals-treated: 149. Data represent the mean ± SEM of three independent biological replicates. A one-way ANOVA and Tukey post-test was used for multiple comparisons. $**p \leq 0.01$; $***p \leq 0.001$. Images shown are z-stack projections. Scale bars, 10 um and 1 um for images and zoom-in, respectively. Unprocessed blots and Source data are provided as a Source Data file.

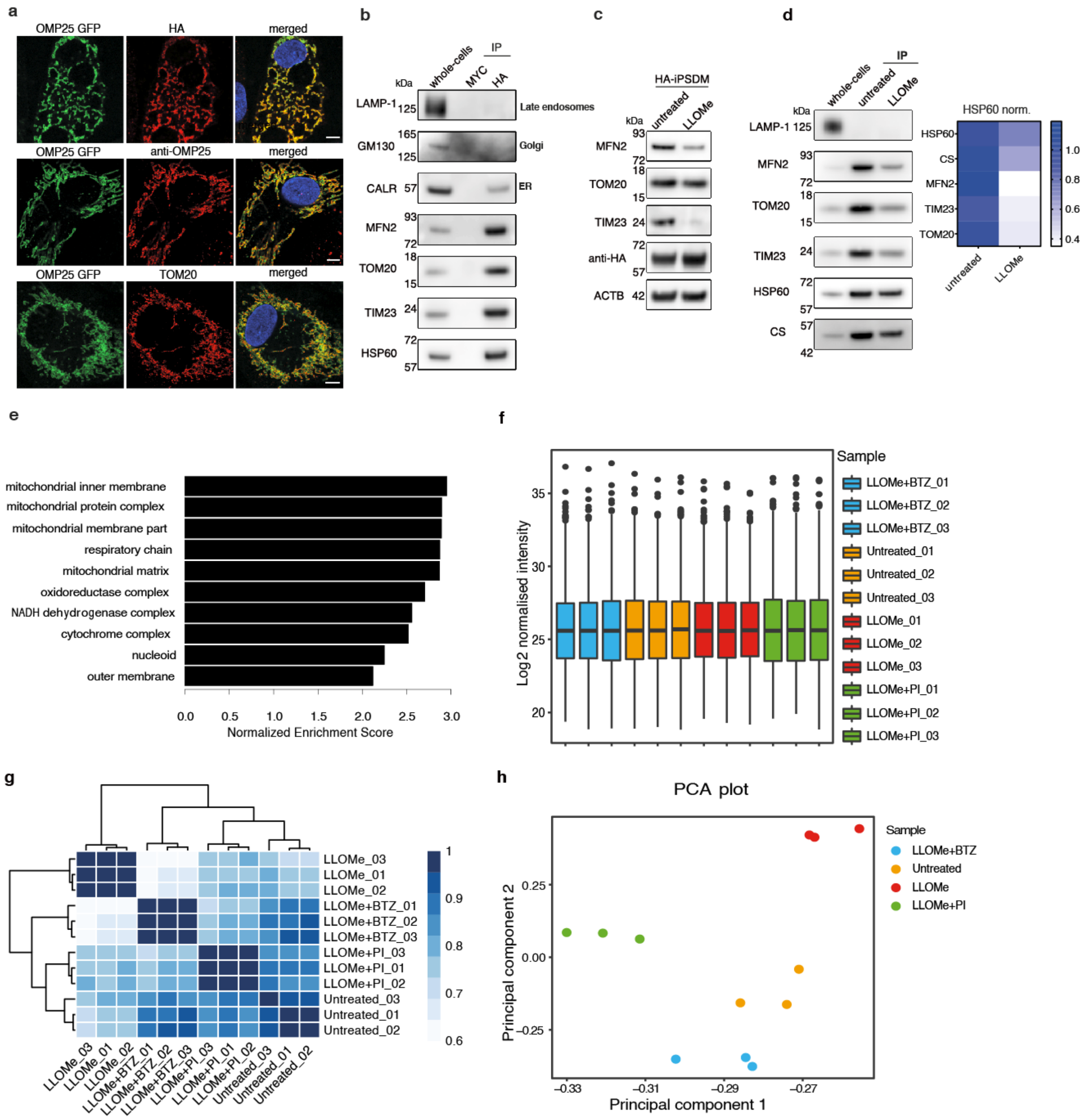

Supplementary Figure 2. Quality control of label-free proteomics data generated from mitochondria isolated by high affinity immunopurification in iPSDM. (a) Representative images of iPSDM expressing 3XHA-EGFP-OMP25 and immunostained with anti-HA, anti-OMP25 and anti-TOM20 specific antibodies n = 25 cells examined per condition. **(b)** Immunoblot for selected organelle markers in iPSDM whole cell lysates or mitochondrial pulldowns using anti-HA beads from iPSDM expressing 3XHA-EGFP-OMP25 or 3XMYC-EGFP-OMP25 (repeated three times with similar results). **(c)** Immunoblot for mitochondrial proteins in iPSDM expressing 3XHA-EGFP-OMP25 treated or not with 0.5 mM LLOMe for 1h (repeated three times with similar results). **(d)** Immunoblot of MITO-tag pulldowns from

iPSDM expressing 3XHA-EGFP-OMP25 treated or not with 0.5 mM LLOMe for 1h. Mitochondrial protein levels were normalized to HSP60 levels from untreated macrophages (heatmap). A whole-cell lysate of untreated iPSDM was run as a control (repeated three times with similar results). **(e)** Gene Set Enrichment Analysis (GSEA) of quantitative proteomics data from HA pulldowns on 3XHA-EGFP-OMP25 versus HA pulldowns on 3xMYC-EGFP-OMP25-expressing iPSDM under control conditions. The 3XMYC-EGFP-OMP25-expressing iPSDM served as a negative control in our analysis, and protein lists were ranked based log_2 fold change. GSEA of GO cellular components was performed using WebGestalt. **(f)** Boxplots of normalised log_2-transformed intensities from mitochondrial immunoprecipitations (Mito-IP) before comparison between treatment groups. Plots visualise the mean, with the box bounds showing the 25th and 75th percentiles and the whiskers the 5th and 95th percentile. **(g)** Correlogram of Pearson correlation coefficients of log_2-transformed intensities from the different treatments and replicates used in the Mito-IP analysis. **(h)** Principal component analysis of log_2-transformed intensities from the different samples used during the Mito-IP analysis. n=3 independent experiments. Scale bars, 10 um. Unprocessed blots and Source data are provided as a Source Data file. See also Supplementary Table 1.

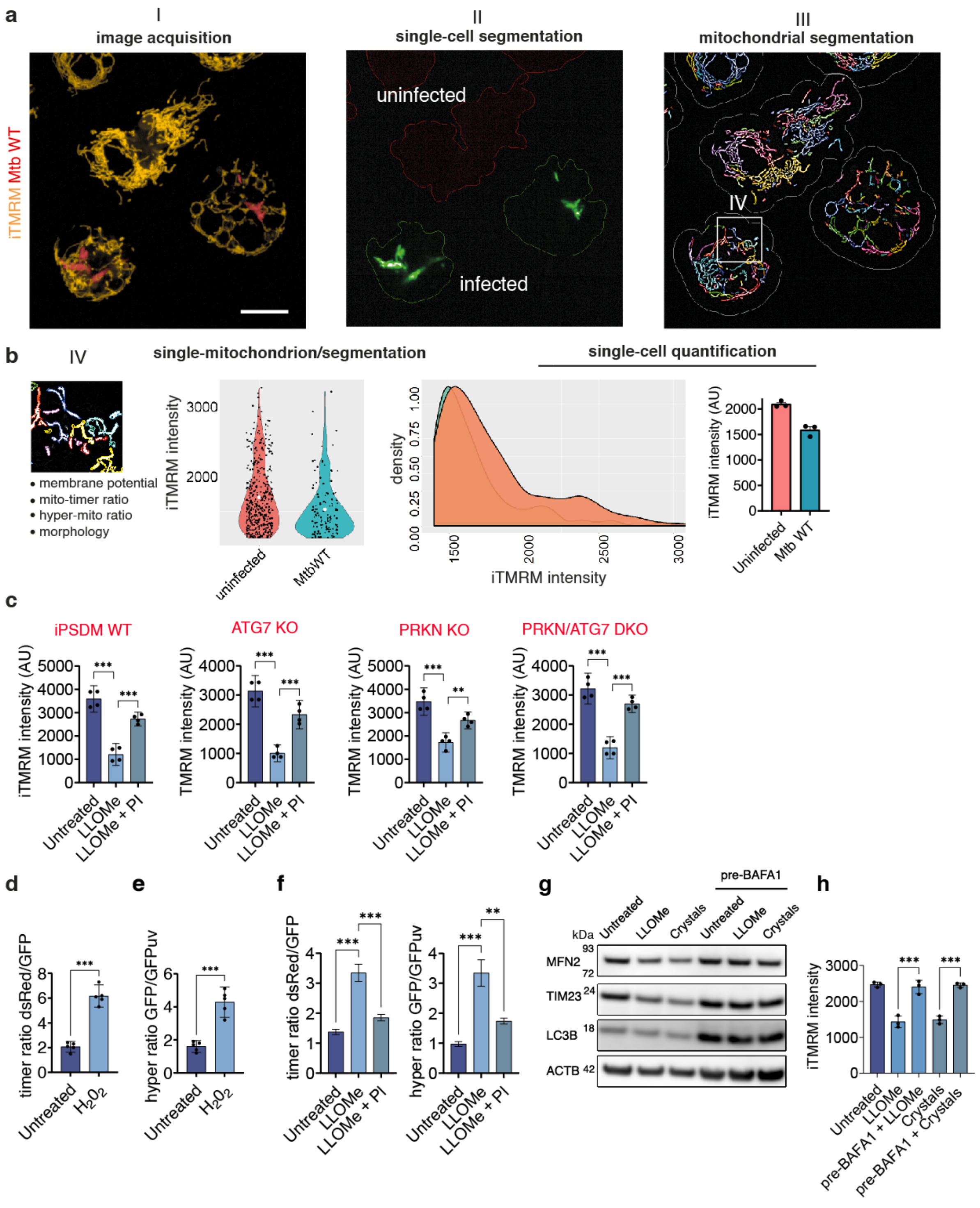

a
I
image acquisition
iTMRM Mtb WT
II
single-cell segmentation
uninfected
infected
III
mitochondrial segmentation
IV
b
IV
• membrane potential
• mito-timer ratio
• hyper-mito ratio
• morphology
single-mitochondrion/segmentation
iTMRM intensity
uninfected
MtbWT
single-cell quantification
density
iTMRM intensity
iTMRM intensity (AU)
Uninfected
Mtb WT
c
iPSDM WT
ATG7 KO
PRKN KO
PRKN/ATG7 DKO
iTMRM intensity (AU)
TMRM intensity (AU)
Untreated
LLOMe
LLOMe + PI
d
timer ratio dsRed/GFP
Untreated
H_2O_2
e
hyper ratio GFP/GFPuv
f
timer ratio dsRed/GFP
hyper ratio GFP/GFPuv
g
pre-BAFA1
kDa
Untreated
LLOMe
Crystals
MFN2
TIM23
LC3B
ACTB
h
iTMRM intensity
pre-BAFA1 + LLOMe
pre-BAFA1 + Crystals

Supplementary Figure 3. Supplementary data related to Figures 3. (a) Representative segmentation strategy using Harmony software (I to IV) in iPSDM is shown to illustrate the mitochondrial analysis pipeline using single-cell high-content imaging. Images shown are z-stack projections. **(b)** Parameters measured by the high content approach and representation of the mitochondrial heterogeneity per condition and intensity distribution in cells. (**c**) Quantification of iTMRM intensity in iPSDM WT, ATG7 KO, PRKN KO and PRKN/ATG7 DKO. Data represent the mean ± SEM of three independent biological replicates. One-way ANOVA and Tukey post-test was used for multiple comparisons. (**d**) iPSDM expressing mitoTimer were treated or not with 100 uM H_2O_2 for 2 h. The dsRed/GFP ratios were quantified by high-content imaging. (**e**) iPSDM expressing Hyper-Mito (pHyPer-dMito) were treated or not with 100 uM H_2O_2 for 1h. The GFPuv/GFP ratios were quantified by high-content imaging. At least 300 cells were counted per condition. An unpaired two-tail t-test test was used for comparisons. **(f)** iPSDM expressing mitoTimer or Hyper-Mito were left untreated or treated with LLOMe (0.5 mM, 1h) and incubated in the presence or absence of a protease inhibitor (PI). **(g,h)** iPSDM were incubated in the presence or the absence of Bafilomycin A1 (BAFA1) (100 nM, 2h) and treated with LLOMe (0.5 mM, 1h) or silica crystals (100 ug/mL, 3h). Mitochondrial protein levels were evaluated by WB (g) and iTMRM (h) intensity evaluated by high-content single-cell microscopy. Data represent mean ± SEM of three independent biological experiments. $^{**}p \leq 0.01$; $^{***}p \leq 0.001$. Images shown are z-stack projections. Scale bars, 10 um. Unprocessed blots and Source data are provided as a Source Data file.

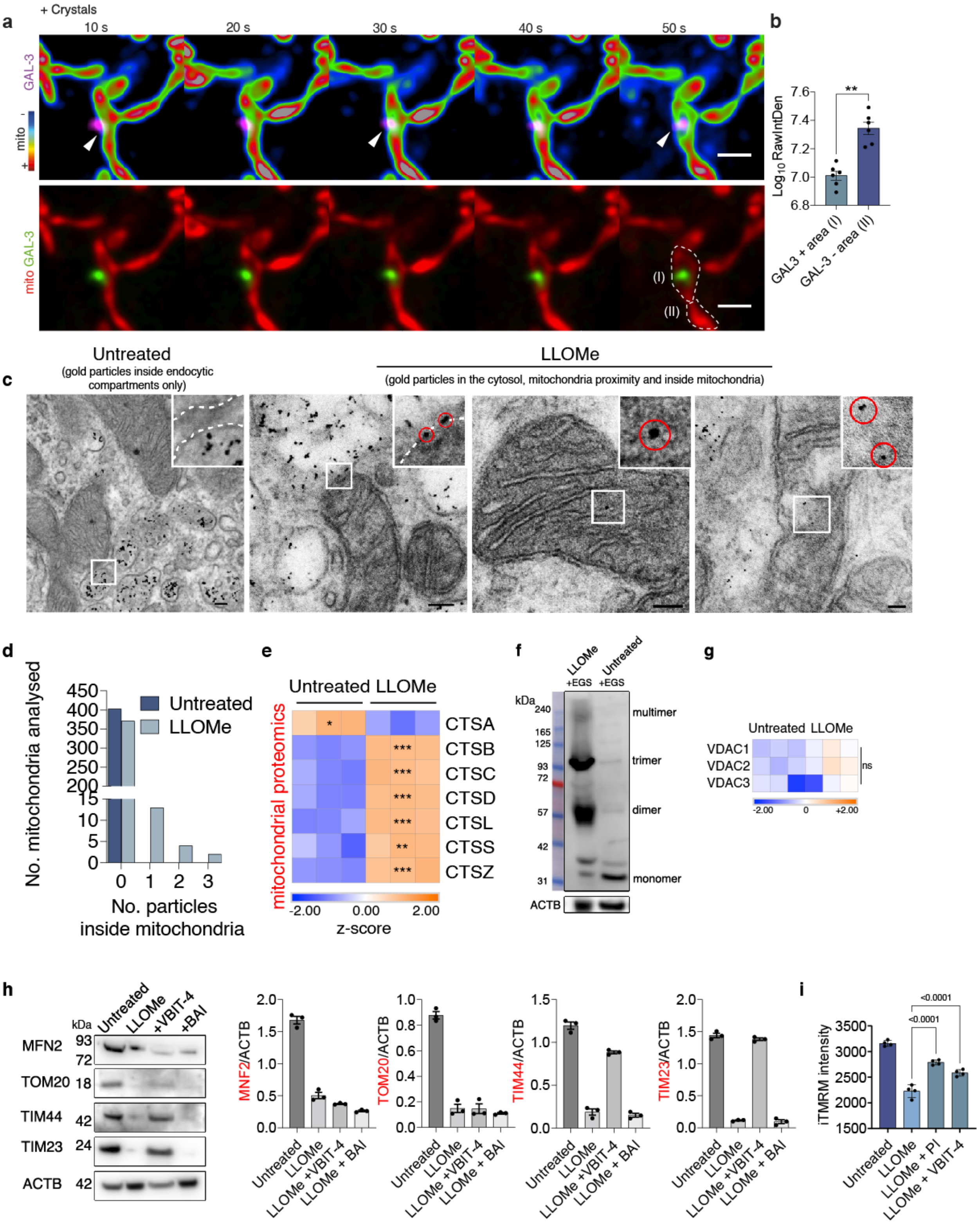

Supplementary Figure 4. Supplementary data related to Figure 4.

(a) iPSDM expressing GAL-3-RFP and incubated with MitoTracker Deep Red were treated with 100ug/mL of silica crystals and imaged immediately after stimulation at 1 frame per 10 s. A selected sequence showing a GAL-3 positive vesicle in proximity of mitochondria is shown. **(b)** MitoTracker Deep Red intensity quantification of mitochondrial areas in contact with GAL-

3- positive vesicles or without interaction (GAL-3-negative), illustrated as “I” and “II”, respectively. Bar plots show data mean values +/- SEM from one out of three independent experiments with n = 12 events per condition. **(c)** Representative electron micrographs of iPSDM incubated with 5nm gold particles and left untreated or treated with 0.5 mM of LLOMe for 1 h. n = 88 cells examined per condition over three independent experiments. **(d)** Quantification of nanogold particles detected per mitochondrion in the indicated conditions. **(e)** Heatmap indicating z-score values of lysosomal cathepsins significantly increased in the MITO-tag pulldown from iPSDM untreated or treated with LLOMe. **(f)** VDAC oligomerisation evaluated by WB in iPSDM left untreated or treated with LLOMe (0.5 mM, 1h). **(g)** Heatmap with z-score values showing VDAC1, VDAC2 and VDAC3 protein levels. **(h)** mitochondrial protein levels of iPSDM pre-incubated with VBIT-4 (10 μM, 6h) or BAI (2 μM, 6 h). Bar plots show the respective protein levels relative to ACTB from three independent experiments. **(i)** iTMRM intensity levels in iPSDM left untreated or treated with LLOMe and incubated with VBIT-4 or with a protease inhibitor cocktail (PI). Unprocessed blots and Source data are provided as a Source Data file.

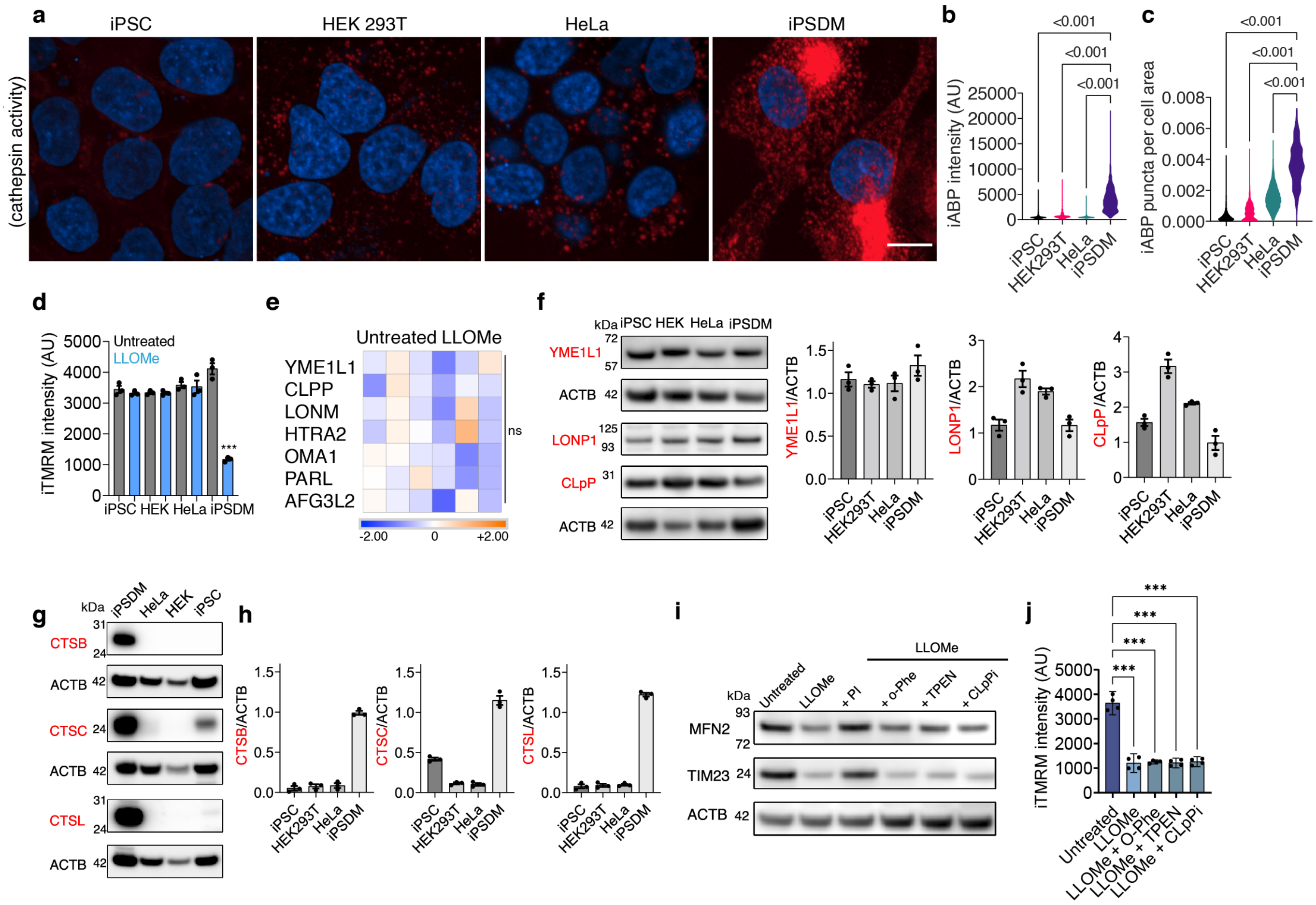

Supplementary Figure 5. Supplementary data related to Figure 4.
(a) representative images of iPSC, HEK293T, HeLa cells and iPSDM incubated for 3h with the cathepsin activity-based probe iABP (1uM), n = 3 independent experiments with at least 300 cells evaluated per condition. **(b, c)** high-content single-cell quantification of iABP lysosomal intensity (b) and iABP puncta per area of cell (c). Results are representative of one out of three independent experiments with at least 300 cells evaluated per condition. One-way ANOVA and Tukey post-test was used for multiple comparisons. **(d)** iTMRM intensity evaluation in the indicated cell types after 6h of LLOMe 1mM (iPSC, HEK293T, HeLa) or after 1h of LLOMe 0.5mM (iPSDM). **(e)** Heatmap with z-score values showing mitoproteases protein levels. **(f)** YME1L1, LONP1 and CLpP protein levels were evaluated by WB in iPSDM, HeLa, HEK293T and iPSC. Bar plots show protein levels relative to ACTB, n = 3 independent experiments. **(g)** active cathepsin B, C and L protein levels were evaluated by WB in iPSDM, HeLa, HEK293T and iPSC **(h)** bar plots show protein levels relative to ACTB, n = 3 independent experiments. **(i, j)** iPSDM incubated in the presence or the abscence of the selective mitochondrial protease inhibitors 1,10-phenanthroline (o-Phe) (1mM, 6 h), TPEN (0.2 mM, 6 h) or A2-32-01 (CLpPi) (50 um, 6 h) and treated with LLOMe (0.5mM, 1 h). The protease inhibitor (PI) treatment was done simultaneously with LLOMe as described before. After that, mitochondrial protein levels were evaluated by WB (i) and iTMRM intensity analysed by high-content single-cell microscopy (j). Bar plots represent mean ± SEM of three

independent biological experiments. Unprocessed blots and Source data are provided as a Source Data file.

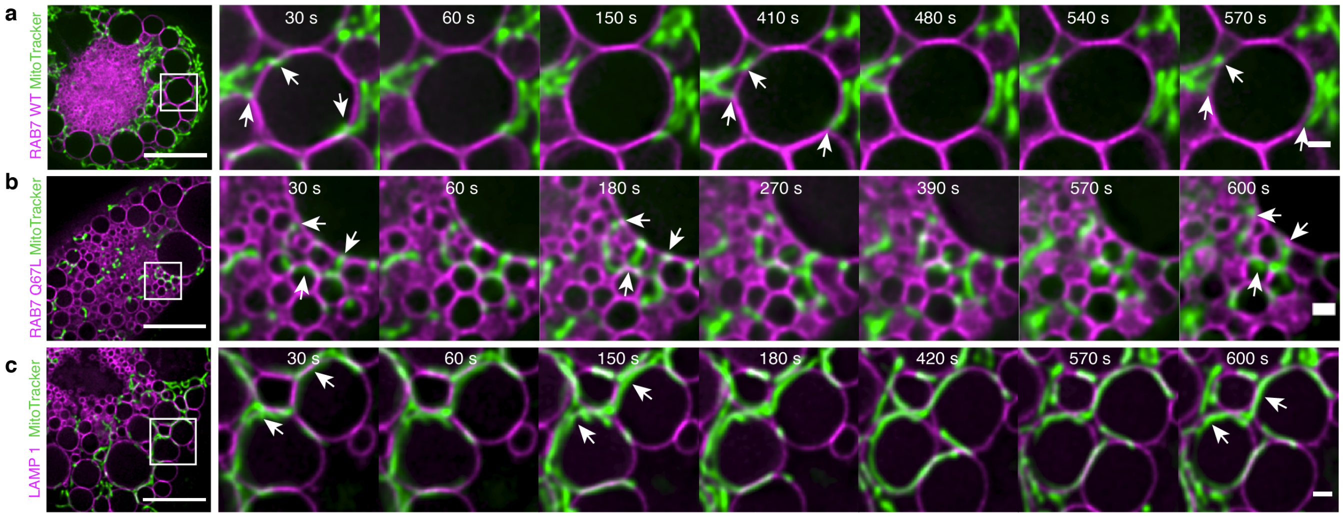

Supplementary Figure 6. Supplementary data related mitochondria-lysosome interaction analysis shown in Figure 4.

(a-c) Live-cell super-resolution imaging (30 s time frame) of iPSDM transiently expressing RAB7 WT GFP (a), RAB(Q67L) GFP (b) or LAMP1-mNeonGreen (c) and incubated with MitoTracker Deep Red. Arrows indicate mitochondria (M) - lysosome (L) contacts. Scale bars: 10 µm and 1 µm for images and zoom-in, respectively. Source data are provided as a Source Data file.

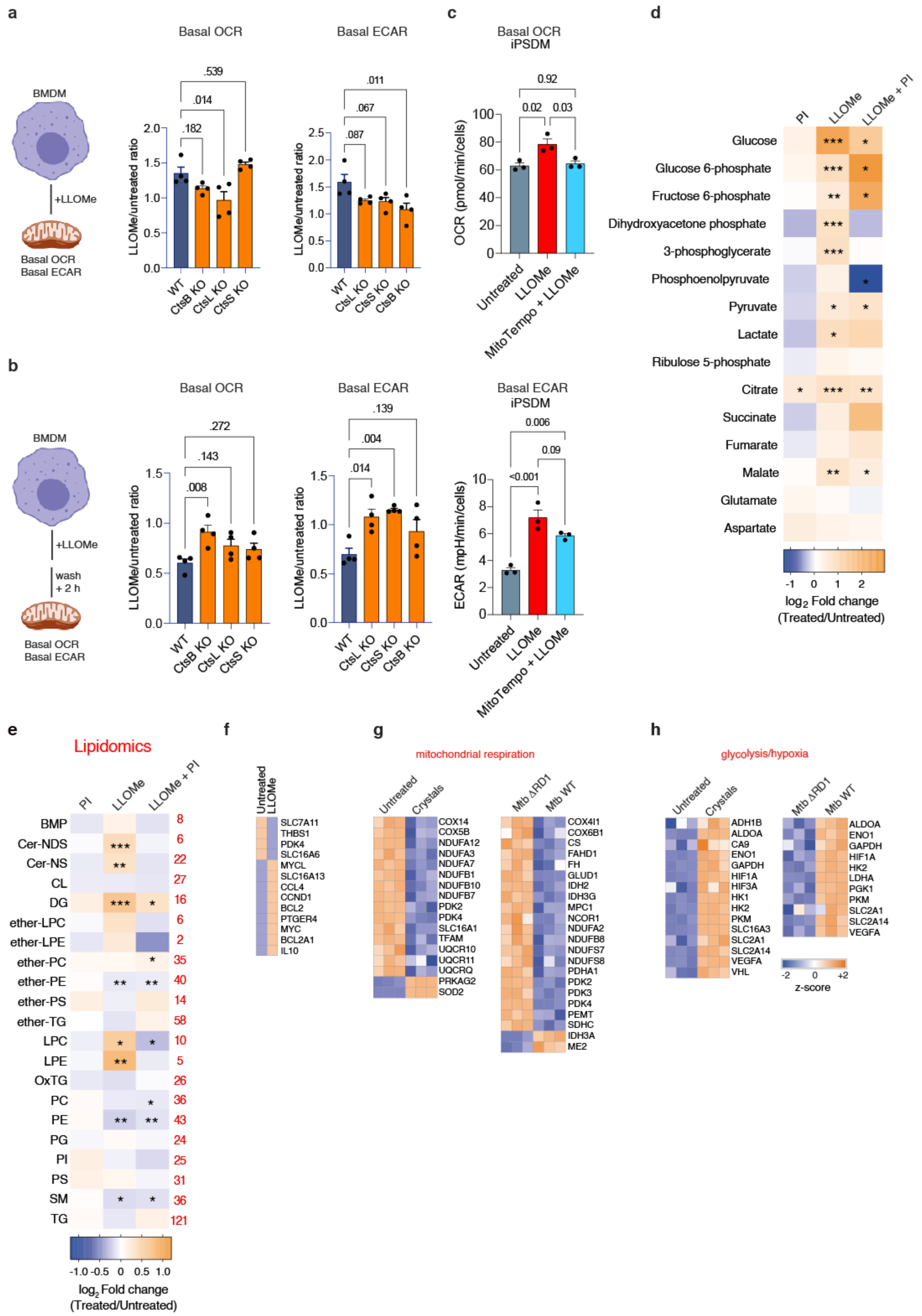

Supplementary Figure 7. Lysosomal leakage affects macrophage metabolism.

(**a**) Bar graphs show the ratio LLOMe vs untreated of basal OCR and ECAR values of BMDM WT, CtsB KO, CtsL KO and CtsS KO stimulated with 0.5 mM of LLOMe for 1 h. **(b)** BMDM

were treated as in A but ECAR and OCR evaluation started after 2h of removing LLOMe treatment. Data represent the mean ± SEM of two out of three independent biological replicates. Values were normalised to cell number. A one-way ANOVA and Dunnett post-test were used for multiple comparisons vs BMDM WT. **(c)** Bar graphs show the basal OCR and ECAR levels of iPSDM pre-treated with Mito-Tempo (5 uM , 1h)and left untreated or treated with 0.5 mM of LLOMe for 1 h. A one-way ANOVA and Tukey post-test were used for multiple comparisons. Values were normalised to cell number. **(d, e)** Total metabolite abundance **(d)** and lipidomics **(e)** of iPSDM untreated or treated with 0.5 mM of LLOMe for 1 h in the presence or absence of PI. Values indicate $\log_2$ fold-change relative to untreated iPSDM. The number of lipids per class is indicated in red. Student's t test, n = 5 technical replicates. **(f)** Gene expression analysis using NanoString of iPSDM untreated or treated with 0.5 mM LLOMe for 1 h **(g, h)** Metabolism-related gene expression analysis using NanoString showing mitochondrial respiration- (g) or glycolysis- (h) related genes of iPSDM untreated or treated with 100 ug/mL silica crystals for 4 h or infected with Mtb WT or Mtb ΔRD1for 48 h. Genes with $\log_2$ fold change >1 and $p \leq 0.05$ are shown and heatmaps indicate z-score values. Data is from one representative experiment with three technical replicates. BMP: bis(monoacylglycero)phosphate, Cer-NDS: ceramide-NDS, Cer-NS: ceramide-NS, CL: cardiolipin, DG: diacylglycerol, ether-LPC: ether-lysophosphatidylcholine, ether-LPE: ether-lysophosphatidylethanolamine, ether-PC: ether-phosphatidylcholine, ether-PE: ether-phosphatidylethanolamine, ether-PS: ether-phosphatidylserine, ether-TG: ether-triacylglycerol, LPC: lysophosphatidylcholine, LPE: lysophosphatidylethanolamine, OxTG: Oxidised Triacylglycerol, PC: phosphatidylcholine, PE: phosphatidylethanolamine, PG: phosphatidylglycerol, PI: phosphatidylinositol, PS: phosphatidylserine, SM: sphingomyelin, TG: triacylglycerol. Source data are provided as a Source Data file. See also Supplementary Table 3.

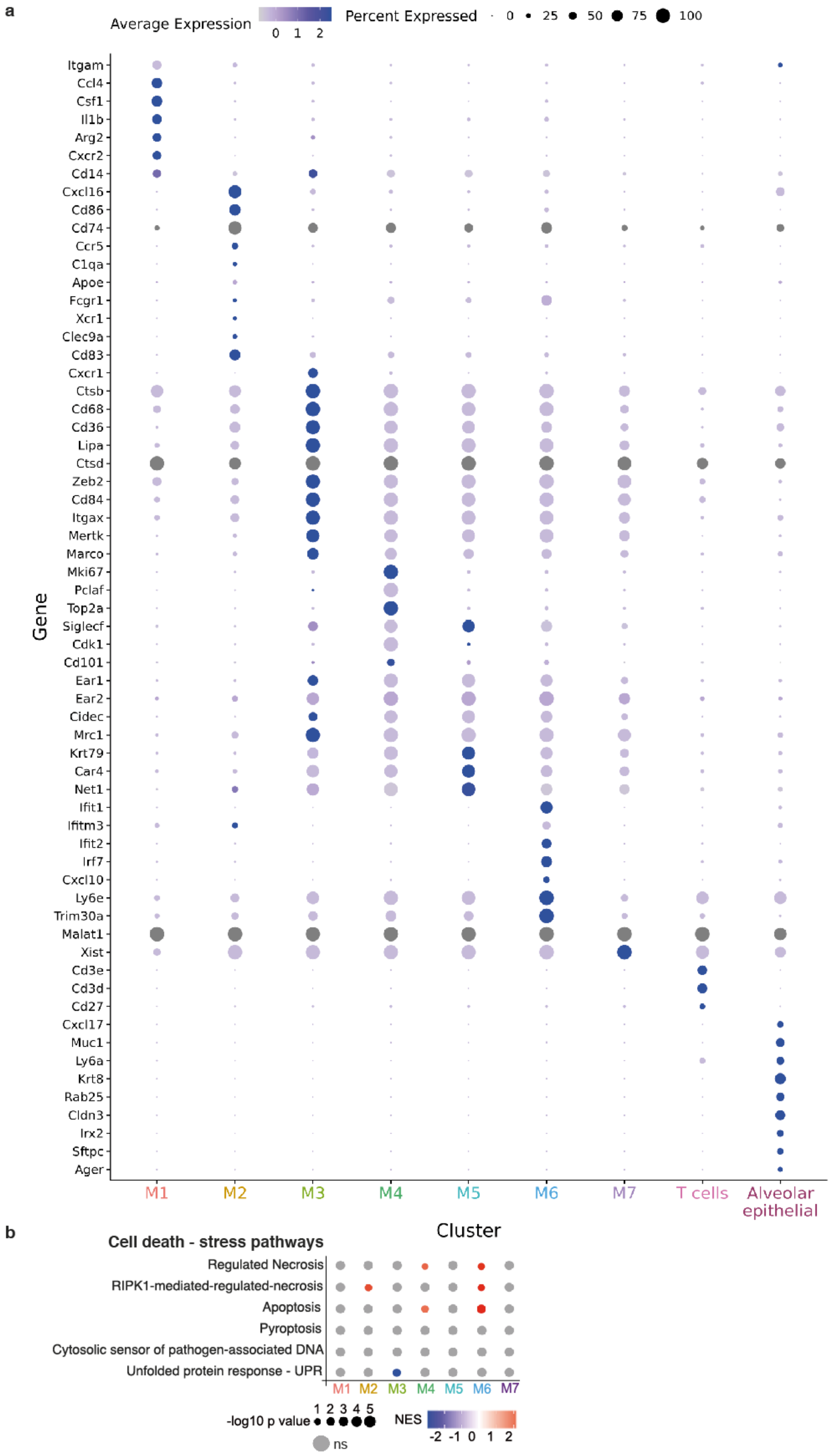
a
Average Expression
0 1 2
Percent Expressed
0 25 50 75 100
Gene
Itgam
Ccl4
Csf1
Il1b
Arg2
Cxcr2
Cd14
Cxcl16
Cd86
Cd74
Ccr5
C1qa
Apoe
Fcgr1
Xcr1
Clec9a
Cd83
Cxcr1
Ctsb
Cd68
Cd36
Lipa
Ctsd
Zeb2
Cd84
Itgax
Mertk
Marco
Mki67
Pclaf
Top2a
Siglecf
Cdk1
Cd101
Ear1
Ear2
Cidec
Mrc1
Krt79
Car4
Net1
Ifit1
Ifitm3
Ifit2
Irf7
Cxcl10
Ly6e
Trim30a
Malat1
Xist
Cd3e
Cd3d
Cd27
Cxcl17
Muc1
Ly6a
Krt8
Rab25
Cldn3
Irx2
Sftpc
Ager
M1
M2
M3
M4
M5
M6
M7
T cells
Alveolar epithelial
Cluster
b
Cell death - stress pathways
Regulated Necrosis
RIPK1-mediated-regulated-necrosis
Apoptosis
Pyroptosis
Cytosolic sensor of pathogen-associated DNA
Unfolded protein response - UPR
M1
M2
M3
M4
M5
M6
M7
-log10 p value
1 2 3 4 5
ns
NES
-2 -1 0 1 2

Supplementary Figure 8- Single cell RNA-seq analysis of the lung macrophage subsets after endomembrane damage. (a) Dot-plot showing expression levels of representative genes for each cluster **(b)** Cell death-related pathways significantly enriched by the treatment among the different macrophage populations. Source data are provided in Supplementary Table 5 and 6.

See also https://shiny.crick.ac.uk/033_scrnaseq_airspace_cells_inflammation/865eb86c8eca0/

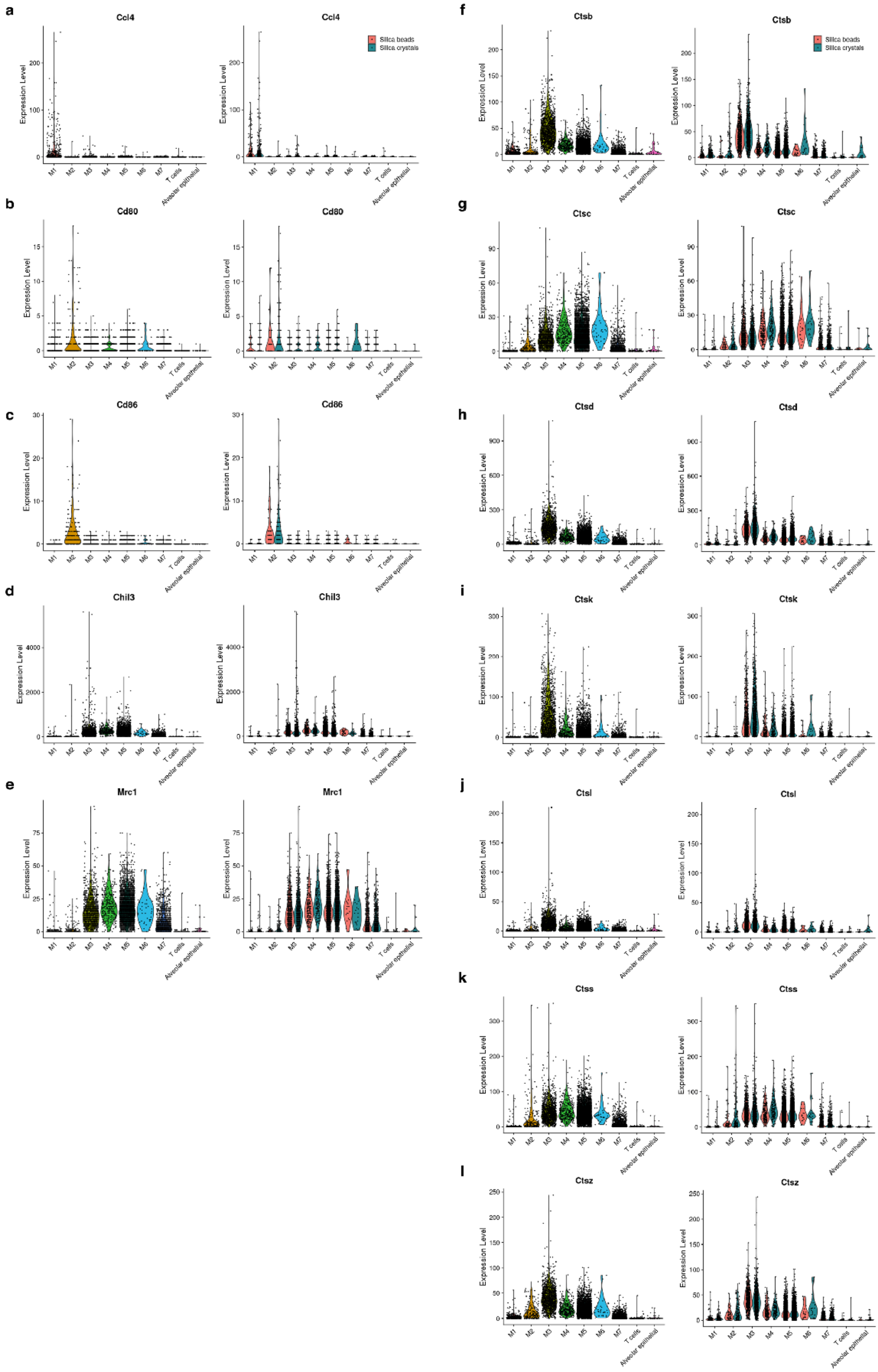
a
Ccl4
b
Cd80
c
Cd86
d
Chil3
e
Mrc1
f
Ctsb
g
Ctsc
h
Ctsd
i
Ctsk
j
Ctsl
k
Ctss
l
Ctsz
Silica beads
Silica crystals
Expression Level
M1
M2
M3
M4
M5
M6
M7
T cells
Alveolar epithelial

Supplementary Figure 9. Cathepsins and M1/M2 transcript levels from the single cell RNA-seq dataset of lung macrophage subsets after endomembrane damage. (a-e) violin plots show transcript levels per cluster (left) and experimental condition (right) of the indicated macrophage polarisation markers. **(f-l)** violin plots show transcript levels per cluster (left) and experimental condition (right) of the indicated lysosomal cathepsins. Source data are provided in Supplementary Table 5 and 6.
See also https://shiny.crick.ac.uk/033_scrnaseq_airspace_cells_inflammation/865eb86c8eca0/

Supplementary Table 1. Comparative analysis of mitochondrial processes using the iPSDM MITO-tag dataset.

Heatmap I

Log_2 fold change of mitochondrial proteins involved in oxidative phosphorylation (A) and translation in mitochondria (B) for each indicated comparison, $p<0.05$. Of note that the overall mitochondrial protein decrease observed after LLOMe treatment is rescued in the presence of a protease inhibitor (PI) (seen as a positive Log_2 fold change value in the LLOMe+PI vs LLOMe comparison). The MITO-tag data was analysed using mitoXplorer 2.0[1] (http://mitoxplorer2.ibdm.univ-mrs.fr/)

Heatmap II

Log_2 fold change of mitochondrial proteins involved in oxidative phosphorylation (A) and translation in mitochondria (B) for each indicated comparison, $p<0.05$. Of note that the overall mitochondrial protein decrease observed after LLOMe treatment is rescued in the presence of a protease inhibitor (PI) (seen as a positive Log_2 fold change value in the LLOMe+PI vs LLOMe, and Untreated vs LLOMe comparisons). The MITO-tag data was analysed using mitoXplorer 2.0 (http://mitoxplorer2.ibdm.univ-mrs.fr/)

1. Marchiano, F., Haering, M. & Habermann, B.H. The mitoXplorer 2.0 update: integrating and interpreting mitochondrial expression dynamics within a cellular context. Nucleic Acids Res (2022).

Heatmap I

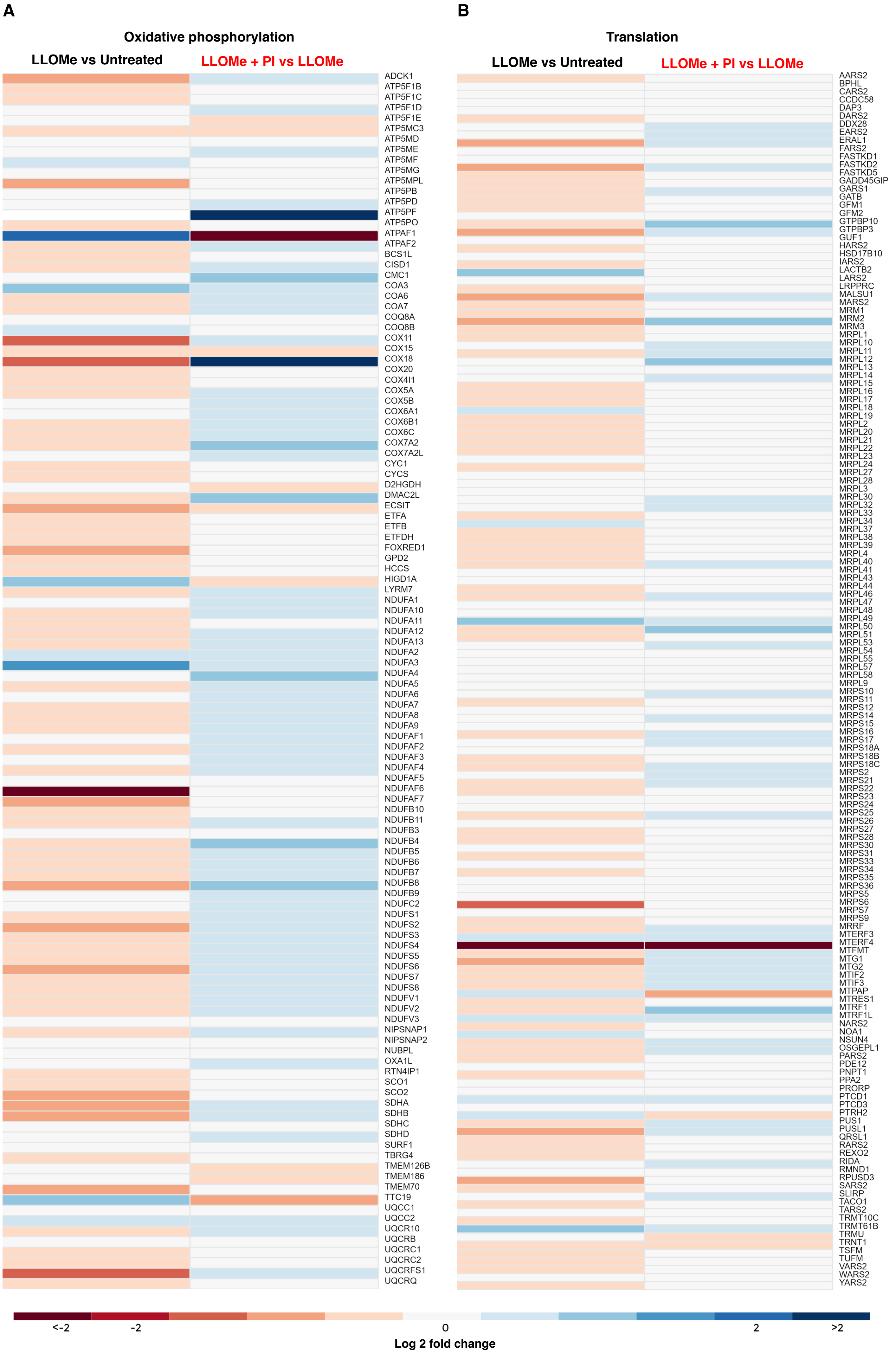
A
Oxidative phosphorylation
LLOMe vs Untreated
LLOMe + PI vs LLOMe
ADCK1
ATP5F1B
ATP5F1C
ATP5F1D
ATP5F1E
ATP5MC3
ATP5MD
ATP5ME
ATP5MF
ATP5MG
ATP5MPL
ATP5PB
ATP5PD
ATP5PF
ATP5PO
ATPAF1
ATPAF2
BCS1L
CISD1
CMC1
COA3
COA6
COA7
COQ8A
COQ8B
COX11
COX15
COX18
COX20
COX4I1
COX5A
COX5B
COX6A1
COX6B1
COX6C
COX7A2
COX7A2L
CYC1
CYCS
D2HGDH
DMAC2L
ECSIT
ETFA
ETFB
ETFDH
FOXRED1
GPD2
HCCS
HIGD1A
LYRM7
NDUFA1
NDUFA10
NDUFA11
NDUFA12
NDUFA13
NDUFA2
NDUFA3
NDUFA4
NDUFA5
NDUFA6
NDUFA7
NDUFA8
NDUFA9
NDUFAF1
NDUFAF2
NDUFAF3
NDUFAF4
NDUFAF5
NDUFAF6
NDUFAF7
NDUFB10
NDUFB11
NDUFB3
NDUFB4
NDUFB5
NDUFB6
NDUFB7
NDUFB8
NDUFB9
NDUFC2
NDUFS1
NDUFS2
NDUFS3
NDUFS4
NDUFS5
NDUFS6
NDUFS7
NDUFS8
NDUFV1
NDUFV2
NDUFV3
NIPSNAP1
NIPSNAP2
NUBPL
OXA1L
RTN4IP1
SCO1
SCO2
SDHA
SDHB
SDHC
SDHD
SURF1
TBRG4
TMEM126B
TMEM186
TMEM70
TTC19
UQCC1
UQCC2
UQCR10
UQCRB
UQCRC1
UQCRC2
UQCRFS1
UQCRQ
B
Translation
LLOMe vs Untreated
LLOMe + PI vs LLOMe
AARS2
BPHL
CARS2
CCDC58
DAP3
DARS2
DDX28
EARS2
ERAL1
FARS2
FASTKD1
FASTKD2
FASTKD5
GADD45GIP
GARS1
GATB
GFM1
GFM2
GTPBP10
GTPBP3
GUF1
HARS2
HSD17B10
IARS2
LACTB2
LARS2
LRPPRC
MALSU1
MARS2
MRM1
MRM2
MRM3
MRPL1
MRPL10
MRPL11
MRPL12
MRPL13
MRPL14
MRPL15
MRPL16
MRPL17
MRPL18
MRPL19
MRPL2
MRPL20
MRPL21
MRPL22
MRPL23
MRPL24
MRPL27
MRPL28
MRPL3
MRPL30
MRPL32
MRPL33
MRPL34
MRPL37
MRPL38
MRPL39
MRPL4
MRPL40
MRPL41
MRPL43
MRPL44
MRPL46
MRPL47
MRPL48
MRPL49
MRPL50
MRPL51
MRPL53
MRPL54
MRPL55
MRPL57
MRPL58
MRPL9
MRPS10
MRPS11
MRPS12
MRPS14
MRPS15
MRPS16
MRPS17
MRPS18A
MRPS18B
MRPS18C
MRPS2
MRPS21
MRPS22
MRPS23
MRPS24
MRPS25
MRPS26
MRPS27
MRPS28
MRPS30
MRPS31
MRPS33
MRPS34
MRPS35
MRPS36
MRPS5
MRPS6
MRPS7
MRPS9
MRRF
MTERF3
MTERF4
MTFMT
MTG1
MTG2
MTIF2
MTIF3
MTPAP
MTRES1
MTRF1
MTRF1L
NARS2
NOA1
NSUN4
OSGEPL1
PARS2
PDE12
PNPT1
PPA2
PRORP
PTCD1
PTCD3
PTRH2
PUS1
PUSL1
QRSL1
RARS2
REXO2
RIDA
RMND1
RPUSD3
SARS2
SLIRP
TACO1
TARS2
TRMT10C
TRMT61B
TRMU
TRNT1
TSFM
TUFM
VARS2
WARS2
YARS2
<-2
-2
0
2
>2
Log 2 fold change

Heatmap II

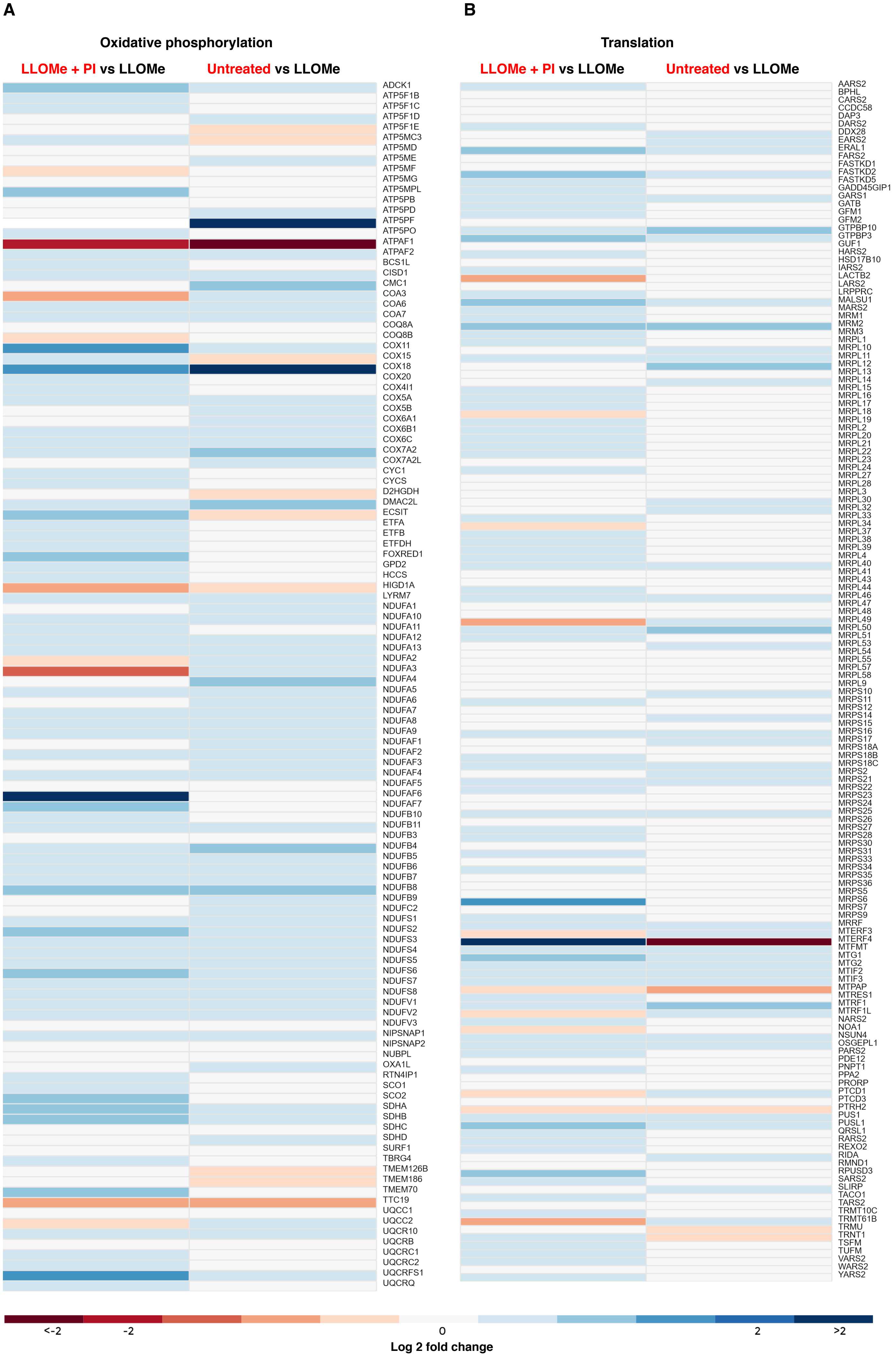

A
Oxidative phosphorylation
LLOMe + PI vs LLOMe
Untreated vs LLOMe
ADCK1
ATP5F1B
ATP5F1C
ATP5F1D
ATP5F1E
ATP5MC3
ATP5MD
ATP5ME
ATP5MF
ATP5MG
ATP5MPL
ATP5PB
ATP5PD
ATP5PF
ATP5PO
ATPAF1
ATPAF2
BCS1L
CISD1
CMC1
COA3
COA6
COA7
COQ8A
COQ8B
COX11
COX15
COX18
COX20
COX4I1
COX5A
COX5B
COX6A1
COX6B1
COX6C
COX7A2
COX7A2L
CYC1
CYCS
D2HGDH
DMAC2L
ECSIT
ETFA
ETFB
ETFDH
FOXRED1
GPD2
HCCS
HIGD1A
LYRM7
NDUFA1
NDUFA10
NDUFA11
NDUFA12
NDUFA13
NDUFA2
NDUFA3
NDUFA4
NDUFA5
NDUFA6
NDUFA7
NDUFA8
NDUFA9
NDUFAF1
NDUFAF2
NDUFAF3
NDUFAF4
NDUFAF5
NDUFAF6
NDUFAF7
NDUFB10
NDUFB11
NDUFB3
NDUFB4
NDUFB5
NDUFB6
NDUFB7
NDUFB8
NDUFB9
NDUFC2
NDUFS1
NDUFS2
NDUFS3
NDUFS4
NDUFS5
NDUFS6
NDUFS7
NDUFS8
NDUFV1
NDUFV2
NDUFV3
NIPSNAP1
NIPSNAP2
NUBPL
OXA1L
RTN4IP1
SCO1
SCO2
SDHA
SDHB
SDHC
SDHD
SURF1
TBRG4
TMEM126B
TMEM186
TMEM70
TTC19
UQCC1
UQCC2
UQCR10
UQCRB
UQCRC1
UQCRC2
UQCRFS1
UQCRQ
B
Translation
LLOMe + PI vs LLOMe
Untreated vs LLOMe
AARS2
BPHL
CARS2
CCDC58
DAP3
DARS2
DDX28
EARS2
ERAL1
FARS2
FASTKD1
FASTKD2
FASTKD5
GADD45GIP1
GARS1
GATB
GFM1
GFM2
GTPBP10
GTPBP3
GUF1
HARS2
HSD17B10
IARS2
LACTB2
LARS2
LRPPRC
MALSU1
MARS2
MRM1
MRM2
MRM3
MRPL1
MRPL10
MRPL11
MRPL12
MRPL13
MRPL14
MRPL15
MRPL16
MRPL17
MRPL18
MRPL19
MRPL2
MRPL20
MRPL21
MRPL22
MRPL23
MRPL24
MRPL27
MRPL28
MRPL3
MRPL30
MRPL32
MRPL33
MRPL34
MRPL37
MRPL38
MRPL39
MRPL4
MRPL40
MRPL41
MRPL43
MRPL44
MRPL46
MRPL47
MRPL48
MRPL49
MRPL50
MRPL51
MRPL53
MRPL54
MRPL55
MRPL57
MRPL58
MRPL9
MRPS10
MRPS11
MRPS12
MRPS14
MRPS15
MRPS16
MRPS17
MRPS18A
MRPS18B
MRPS18C
MRPS2
MRPS21
MRPS22
MRPS23
MRPS24
MRPS25
MRPS26
MRPS27
MRPS28
MRPS30
MRPS31
MRPS33
MRPS34
MRPS35
MRPS36
MRPS5
MRPS6
MRPS7
MRPS9
MRRF
MTERF3
MTERF4
MTFMT
MTG1
MTG2
MTIF2
MTIF3
MTPAP
MTRES1
MTRF1
MTRF1L
NARS2
NOA1
NSUN4
OSGEPL1
PARS2
PDE12
PNPT1
PPA2
PRORP
PTCD1
PTCD3
PTRH2
PUS1
PUSL1
QRSL1
RARS2
REXO2
RIDA
RMND1
RPUSD3
SARS2
SLIRP
TACO1
TARS2
TRMT10C
TRMT61B
TRMU
TRNT1
TSFM
TUFM
VARS2
WARS2
YARS2
<-2
-2
0
2
>2
Log 2 fold change

Supplementary Table 2. References used to annotate the main clusters identified by Single cell RNA sequencing of BAL samples.

Cluster	Representative genes	Reference
M1	Itgam, Ccl4, Csf1, Il1b, Arg2, Cxcr2, Cd14	[1-3]
M2	Cxcl16, Cd86, Cd74, Ccr5, C1qa, Apoe, Fcgr1, Xcr1, Clec9a, Cd83	[1, 3]
M3	Cxcr1, Ctsb, Cd68, Cd36, Lipa, Ctsd, Zeb2, Cd84, Itgax, Mertk, Marco	[2-4]
M4	Mki67, Pclaf, Top2a, SiglecF, Cdk1, Cd101, Marco	[1, 5]
M5	SiglecF, Ear1, Ear2, Cidec, Mrc1, Krt79, Car4, Net1, Marco	[2, 3, 5]
M6	Ifit1, Ifitm3, Ifit2, Irf7, Cxcl10, Ly6e, Trim30a	[2]
M7	Xist, Zeb2, Mrc1	[2, 6-8]
Alveolar epithelial	Cxcl17, Muc1, Ly6a, Krt8, Cldn3, Irx2, Sfptc, Ager	[5]
T cells	Cd3e, Cd3d, Cd27	[5]

1. Mould, K.J., Jackson, N.D., Henson, P.M., Seibold, M. & Janssen, W.J. Single cell RNA sequencing identifies unique inflammatory airspace macrophage subsets. *JCI Insight* **4** (2019).
2. Scott, C.L. *et al.* The Transcription Factor ZEB2 Is Required to Maintain the Tissue-Specific Identities of Macrophages. *Immunity* **49**, 312-325 e315 (2018).
3. Zilionis, R. *et al.* Single-Cell Transcriptomics of Human and Mouse Lung Cancers Reveals Conserved Myeloid Populations across Individuals and Species. *Immunity* **50**, 1317-1334 e1310 (2019).
4. Huang, S.C. *et al.* Cell-intrinsic lysosomal lipolysis is essential for alternative activation of macrophages. *Nat Immunol* **15**, 846-855 (2014).
5. Travaglini, K.J. *et al.* A molecular cell atlas of the human lung from single-cell RNA sequencing. *Nature* **587**, 619-625 (2020).
6. Wang, H. *et al.* The Long Non-Coding RNA XIST Controls Non-Small Cell Lung Cancer Proliferation and Invasion by Modulating miR-186-5p. *Cell Physiol Biochem* **41**, 2221-2229 (2017).
7. Sun, Y. & Xu, J. TCF-4 Regulated lncRNA-XIST Promotes M2 Polarization Of Macrophages And Is Associated With Lung Cancer. *Onco Targets Ther* **12**, 8055-8062 (2019).
8. Xu, X. *et al.* Silencing of lncRNA XIST inhibits non-small cell lung cancer growth and promotes chemosensitivity to cisplatin. *Aging (Albany NY)* **12**, 4711-4726 (2020).
