# Supplementary material for: Lysosomal damage drives mitochondrial proteome remodelling and reprograms macrophage immunometabolism
Source: Nat Commun. 2022 Nov 28;13:7338. doi: 10.1038/s41467-022-34632-8 (PMC9705561; doi:10.1038/s41467-022-34632-8)
Supplement: Supplementary file 3 — Description of Additional Supplementary Files [file 41467_2022_34632_MOESM3_ESM.pdf]

Description of Additional Supplementary Files

Supplementary Movie 1

Super-resolution time-lapse images of iPSDM incubated with the cathepsin activity-based probe iABP (magenta) and MitoTracker Green (green). Video shows the sequence corresponding to the montages observed in Fig. 4a. The ROIs showing the respective M-L contacts are indicated. Frame interval = 20 s. Scale bar = 5 µm.

Supplementary Movie 2

Super-resolution time-lapse images of iPSDM incubated with the cathepsin activity-based probe iABP (magenta) and MitoTracker Green (green). Video shows the sequence corresponding to the montages observed in Fig. 4b. The ROIs showing the respective M-L contacts are indicated. Frame interval = 20 s. Scale bar = 5 µm.

Supplementary Movie 3

Super-resolution time-lapse images of iPSDM expressing Lamp1-mNeonGreen (magenta), incubated with MitoTracker Deep Red (RGB rainbow scale) and stimulated with LLOMe (0.5 mM). Frame interval = 30 s. Video shows the full sequence corresponding to the cell displayed in Fig. 4n. Scale bar = 10 µm.

Supplementary Movie 4

Super-resolution time-lapse images of iPSDM expressing Lamp1-mNeonGreen (magenta), incubated with MitoTracker Deep Red (RGB rainbow scale) and stimulated with LLOMe (0.5 mM). Frame interval = 30 s. Video shows the sequence corresponding to the montages displayed in Fig. 4k. Scale bar = 5 µm.

Supplementary Movie 5

Super-resolution time-lapse images of iPSDM expressing Lamp1-mNeonGreen (magenta), incubated with MitoTracker Deep Red (RGB rainbow scale) and stimulated with LLOMe (0.5 mM). Frame interval = 30 s. Video shows the sequence corresponding to the montages displayed in Fig. 4l. Scale bar = 5 µm.

Supplementary Data 1.

Mitochondrial proteomics data related to Figure 2 and Supplementary Figure 2.

Supplementary Data 2.

Metabolomics data related to Figure 5 and Supplementary Figure 7.

Supplementary Data 3.

Table shows differentially expressed genes for each population identified in the BAL scRNA-seq dataset, related to Figure 7 and Supplementary Table 2.

Supplementary Data 4.

Table shows Reactome pathway scores for each population identified in the BAL scRNA-seq dataset, related to Figure 7 and Supplementary Figure 8.
