# Supplementary material for: Lysosomal damage drives mitochondrial proteome remodelling and reprograms macrophage immunometabolism
Source: Nat Commun. 2022 Nov 28;13:7338. doi: 10.1038/s41467-022-34632-8 (PMC9705561; doi:10.1038/s41467-022-34632-8)
Supplement: Supplementary file 13 — Reporting Summary [file 41467_2022_34632_MOESM13_ESM.pdf]

nature portfolio

Corresponding author(s): Claudio Bussi and Maximiliano G. Gutierrez

Last updated by author(s): Oct 16, 2022

Reporting Summary

Nature Portfolio wishes to improve the reproducibility of the work that we publish. This form provides structure for consistency and transparency in reporting. For further information on Nature Portfolio policies, see our Editorial Policies and the Editorial Policy Checklist.

Statistics

For all statistical analyses, confirm that the following items are present in the figure legend, table legend, main text, or Methods section.

n/a	Confirmed	
☐	☒	The exact sample size (*n*) for each experimental group/condition, given as a discrete number and unit of measurement
☐	☒	A statement on whether measurements were taken from distinct samples or whether the same sample was measured repeatedly
☐	☒	The statistical test(s) used AND whether they are one- or two-sided<br>*Only common tests should be described solely by name; describe more complex techniques in the Methods section.*
☒	☐	A description of all covariates tested
☐	☒	A description of any assumptions or corrections, such as tests of normality and adjustment for multiple comparisons
☐	☒	A full description of the statistical parameters including central tendency (e.g. means) or other basic estimates (e.g. regression coefficient) AND variation (e.g. standard deviation) or associated estimates of uncertainty (e.g. confidence intervals)
☐	☒	For null hypothesis testing, the test statistic (e.g. *F*, *t*, *r*) with confidence intervals, effect sizes, degrees of freedom and *P* value noted<br>*Give P values as exact values whenever suitable.*
☒	☐	For Bayesian analysis, information on the choice of priors and Markov chain Monte Carlo settings
☒	☐	For hierarchical and complex designs, identification of the appropriate level for tests and full reporting of outcomes
☒	☐	Estimates of effect sizes (e.g. Cohen's *d*, Pearson's *r*), indicating how they were calculated

Our web collection on statistics for biologists contains articles on many of the points above.

Software and code

Policy information about availability of computer code

Data collection	Microscopy images were analysed using Harmony software (Perkin Elmer, version 4.9) , the open source software FIJI/ImageJ or Micro-Manager v 2.0.
Data analysis	Image processing of confocal and electron microscopy images and western blots: FIJI/ImageJ. Image processing and deconvolution of VT-iSIM images were done using Huygens Essential software (Scientific Volume Imaging B.V, Netherlands). Statistical analysis was performed using GraphPad Prism 10 software or R 3.6.3 (The R Project for Statistical Computing). High-content imaging analysis and mean values were obtained using R 3.6.3. The number of biological replicates and the statistical analysis performed and post hoc tests used can be found in the figure legends. All graphs were plotted in GraphPad Prism software, with the exception of the graphs showed in Extended Figure 4 and sc-RNA-seq graphs that were plotted using R 3.6.3.<br>Metabolomics: Data analysis was performed using our in house-developed software MANIC (version 1.0), based on the software package GAVIN. Lipidomics: Qualitative and quantitative analyses were performed using Free Style 1.7 (Thermo Scientific), Progenesis (Nonlinear Dynamics) and LipidMatch.<br>Single-cell datasets analysis: Data was processed using CellRanger v3.0.2 using the prebuilt mm10 index v3.0.0, and individual Seurat objects were created. Data was analyzed with R v4.0.4 (The R Project for Statistical Computing). Posterior analysis was done with Seurat v3.9.9 package. Gene Set Enrichment Analysis (GSEA) was performed with R v4.0.5 (The R Project for Statistical Computing) using the fgsea v1.16.0 package.<br>Mass spectrometry data analysis: All spectra were analysed using MaxQuant 1.6.10.43. Statistical testing was performed in the R statistical programming language using the LIMMA package.Nanostring analysis: nCounter and ROSALIND. Seahorse analysis: WAVE software, version 2.6.1

For manuscripts utilizing custom algorithms or software that are central to the research but not yet described in published literature, software must be made available to editors and reviewers. We strongly encourage code deposition in a community repository (e.g. GitHub). See the Nature Portfolio guidelines for submitting code & software for further information.

Data

Policy information about availability of data

All manuscripts must include a data availability statement. This statement should provide the following information, where applicable:

- Accession codes, unique identifiers, or web links for publicly available datasets
- A description of any restrictions on data availability
- For clinical datasets or third party data, please ensure that the statement adheres to our policy

The metabolomics and lipidomics data has been deposited at Zenodo, under the following DOI: 10.5281/zenodo.5495849. The mass spectrometry proteomics data have been deposited to the ProteomeXchange Consortium via the PRIDE partner repository with the dataset identifier PXD026045. Sc-RNA-seq data is deposited at GEO (accession number GSE174414). All other data needed to evaluate the conclusions of the study are present in the paper or in the supplementary materials. Additional data are available from the corresponding authors upon reasonable request.

Field-specific reporting

Please select the one below that is the best fit for your research. If you are not sure, read the appropriate sections before making your selection.

☒ Life sciences ☐ Behavioural & social sciences ☐ Ecological, evolutionary & environmental sciences

For a reference copy of the document with all sections, see nature.com/documents/nr-reporting-summary-flat.pdf

Life sciences study design

All studies must disclose on these points even when the disclosure is negative.

Sample size	No statistical method was used to predetermine sample size. Standard considerations based on expected variations from previous experiments [10.1126/science.aat9689, 10.15252/embj.2020104494] were applied to determine the necessary repeats to ensure reproducibility and statistical significance. The corresponding number of events that was analyzed is indicated in the Figure legend or Methods section.
Data exclusions	No data were excluded from analyses.
Replication	We have indicated the number of independent experiments performed in the figure legends or the Methods section.
Randomization	No randomization was performed for this study.
Blinding	No blinding was performed for this study.

Reporting for specific materials, systems and methods

We require information from authors about some types of materials, experimental systems and methods used in many studies. Here, indicate whether each material, system or method listed is relevant to your study. If you are not sure if a list item applies to your research, read the appropriate section before selecting a response.

Materials & experimental systems

n/a	Involved in the study	
☐	☒	Antibodies
☐	☒	Eukaryotic cell lines
☒	☐	Palaeontology and archaeology
☐	☒	Animals and other organisms
☒	☐	Human research participants
☒	☐	Clinical data
☒	☐	Dual use research of concern

Methods

n/a	Involved in the study	
☒	☐	ChIP-seq
☒	☐	Flow cytometry
☒	☐	MRI-based neuroimaging

Antibodies

Antibodies used	anti-HSP60, Cell Signalling Technology, Cat#12165S<br>anti-TOM20, Cell Signalling Technology, Cat#42406S<br>anti-ATG7, Cell Signalling Technology, Cat#8558S<br>anti-TIM23, Becton Dickinson, Cat#611222<br>anti-CS Santa Cruz, Cat#sc-390693<br>anti-PRKN, Santa Cruz, Cat#sc-32282

anti-MFN2, Santa Cruz, Cat#sc-515647
Anti-GM130, Santa Cruz , Cat#sc-55591
Anti-Calregulin, Santa Cruz, Cat#sc-166837
anti-MFN2, Abcam, Cat#ab56889
anti-LAMP1, Abcam, Cat#ab24170
anti-HA, Sigma, Cat#H6908
anti-OMP25, Invitrogen, Cat#PA5-51471
anti-TOM20 , Invitrogen , Cat#MA5-34964
Anti-PDH E1 Alpha, Proteintech, Cat#66119-1-Ig
Alexa Fluor 488 anti-mouse/human Mac-2 (Galectin-3), Biolegend, Cat#125410
Brilliant Violet 421™ anti-mouse/human Mac-2 (Galectin-3), Biolegend, Cat#125416
Alexa Fluor 488 anti-mouse F4/80, Biolegend, Cat#123120
HRP-conjugated anti-mouse, Promega, Cat#W4021
HRP-conjugated anti-rabbit, Promega, Cat#W4011

Validation

All the antibodies purchased have been validated in multiple previous studies accessible at the manufacturer's website.

Eukaryotic cell lines

Policy information about cell lines

Cell line source(s)

EIKA2 human iPSCs, Public Health England Culture Collections, Cat#77650059
KOLF2 human iPSCs, Public Health England Culture Collections, Cat#77650100
HEK293A cells, Invitrogen, Cat#R70507
HeLa cells, European Collection of Authenticated Cell Cultures (ECACC), Cat#93021013

Authentication

Authentication results can be accessed at the respective source's website.
https://www.phe-culturecollections.org.uk/products/celllines/generalcell/search.jsp (for EIKA2, KOLF2 and HeLa)
https://www.thermofisher.com/order/catalog/product/R70507#/R70507 (for HEK293A)

Mycoplasma contamination

All cells tested negative for mycoplasma contamination.

Commonly misidentified lines
(See ICLAC register)

Name any commonly misidentified cell lines used in the study and provide a rationale for their use.

Animals and other organisms

Policy information about studies involving animals; ARRIVE guidelines recommended for reporting animal research

Laboratory animals

Mouse: C57BL/6NJ, The Jackson Laboratory – Bred in house
Mouse: C57BL/6NCrl , Charles River Germany, Bred in animal facility Freiburg University
Mouse CtsB-/-; backcrossed > 10 Generations to C57BL/6NCrl, Dr. Thomas Reinheckel, University of Freiburg
Mouse: CtsL-/- ; backcrossed > 10 Generations to C57BL/6NCrl, Dr. Thomas Reinheckel, University of Freiburg
Mouse: CtsS-/-; backcrossed > 10 Generations to C57BL/6NCrl, Dr. Thomas Reinheckel, University of Freiburg
Mice used were from both sexes and eight to ten week-old.

Wild animals

The study did not involve wild animals.

Field-collected samples

The study did not involved samples collected from the field.

Ethics oversight

All protocols for breeding and experiments were approved by the Home Office (U.K.) under project license P4D8F6075 and performed in accordance with the Animal Scientific Procedures Act, 1986.

Note that full information on the approval of the study protocol must also be provided in the manuscript.
